# Selection of Spring Barley Varieties for Exploiting Quantitative Resistance for Powdery Mildew Control

**DOI:** 10.3390/plants15131989

**Published:** 2026-06-27

**Authors:** Antonín Dreiseitl, Marta Zavřelová

**Affiliations:** 1 Department of Integrated Plant Protection, Agrotest Fyto Ltd., CZ-767 01 Kroměříž, Czech Republic; 2 Gene Bank, Department of Plant Genetics and Breeding, Agrotest Fyto Ltd., CZ-767 01 Kroměříž, Czech Republic; zavrelova@vukrom.cz

**Keywords:** spring barley, *Hordeum vulgare*, genebank, powdery mildew, *Blumeria hordei*, detection of major resistance genes, quantitative resistance, mislabeled accessions

## Abstract

Barley (*Hordeum vulgare* L.) is an important cereal, and powdery mildew caused by *Blumeria hordei* is a major disease of this crop. The most appropriate way to protect barley from the disease is to grow resistant varieties, and a method to achieve this is to exploit quantitative minor genes that confer sufficiently effective and long-lasting resistance. However, when searching for quantitative resistance, broken major genes can also play a role and might affect the infection of evaluated varieties, especially on small plots in experimental fields. Therefore, our present research focused on identifying accessions without major resistance genes. We tested 2804 genebank accessions of spring barley originating from 70 countries using nine isolates of the pathogen known to have a very wide spectrum of avirulence. In 143 accessions, no major resistance gene was found, and 304 accessions carried major genes *Mla8* or *MlCh*, which cannot affect their resistance in the field because of the absence of corresponding avirulent pathotypes. These 447 accessions can therefore be preferably used to reveal and exploit targeted minor genes.

## 1. Introduction

**Figure 1:**
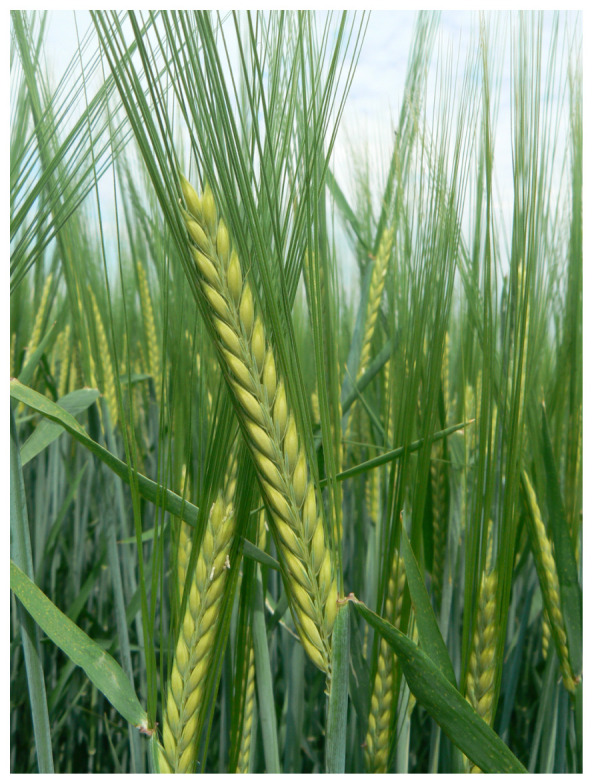
Barley (*Hordeum vulgare* L.).

Barley (*Hordeum vulgare* L.) (Figure 1) is an important cereal grown in 2023 on an area exceeding 46 Mha with a grain production of almost 146 Mt [1]. Powdery mildew (PM), caused by the airborne ascomycete fungus *Blumeria hordei* (M. Liu & Hambl.), is a major disease of this crop. The study of PM on barley was one of the first to verify the validity of the rediscovered Mendelian laws of inheritance relating to plant disease resistance [2]. Subsequent studies of host–pathogen relationships [3] confirmed the proposed gene-for-gene concept [4].

The most appropriate way to protect the crop from the disease is to grow resistant varieties. The deliberate use of genetic resistance to PM in barley breeding was initiated by Honecker [5]. He transferred the *Mlg* gene from the variety Pflugs Intensiv into strains designated Weihenstephaner I and Weihenstephaner II and these were used in the first spring barley varieties bred for PM resistance. Many researchers and breeders subsequently exploited numerous major genes conferring specific PM resistance (R genes) [6,7] and generated hundreds of varieties over several decades [8,9]. However, a general limitation of R genes is their short period of effectiveness, because of the extreme adaptability of the pathogen [10,11,12]. This results in a typical ‘boom-and-bust’ cycle, in which an expansion in the cultivation area of varieties carrying new and effective R genes is soon followed by a breakdown of their resistance usually resulting in a rapid reduction of their agricultural use [13].

A new broad-spectrum, recessively inherited monogenic resistance to PM–Mlo—was first recognized in an induced mutant M66 derived from Haisa [14]. The same resistance was subsequently discovered in other induced barley mutants and in landraces collected in Ethiopia [15] and was designated as a new kind of resistance [6]. The first spring barley variety, Atem, possessing Mlo, was registered in the Netherlands in 1979, and was soon followed by many other varieties [16], particularly once the durability of this resistance had been predicted [17].

An example of the increase in the number of European spring barley varieties carrying Mlo is provided by data from the Czech Republic (central Europe). On average, about five new varieties are released each year, and the total number of registered varieties is about 60–70. The first commercial variety possessing Mlo–Forum—was released in 1993. Up to the end of 2023, 164 varieties originating from nine central and western European countries were registered, 114 of which possess Mlo. In 1993–1995, only one (Forum) of eight newly registered varieties contained Mlo, whereas in 2021–2023, 22 out of 23 varieties possessed this durable resistance [18]; the last specific resistance (SI-I) was introduced in Zeppelin, registered in 2012 [9].

Regarding the current situation, two conclusions can be drawn: (i) specific resistances have not proven themselves since their effectiveness is short because of the rapid adaptation of the pathogen, and (ii) the PM resistance of current European spring barley varieties has focused on a single gene, *mlo*. This “Mlo monopoly” is contrary to the general requirement for diversity of resistances used in the breeding of commercial varieties. Moreover, there are alarming indications that the pathogen might be able to adapt even to this durable resistance [19,20].

An alternative approach might focus on the quantitative resistance of the crop to the pathogen [21,22]. Individual minor genes of non-specific resistance contribute less to the varietal resistance than efficient major genes; they can confer reduced infection rather than immunity to the disease [23,24], but their accumulation (pyramiding) can provide effective and long-lasting resistance [25]. However, when searching for quantitative resistance genes, qualitative major genes also play a part and can affect the infection of evaluated varieties in the field, especially broken genes on small experimental plots [26,27,28]. Therefore, our present research focused on the detection of major resistance genes in a large set of varieties. Varieties carrying major resistance genes, including those that have a slight effect that can be easily confused with the efficacy of minor genes, can be excluded from subsequent evaluations targeting minor genes.

## 2. Results

Two thousand eight hundred and four GB spring barley accessions were separately inoculated with nine selected PM isolates, and major resistance genes (mostly specific) were detected. Table S1 comprises a list of accessions (varieties), accession numbers, the DOI numbers of registered, mostly commercial domestic varieties (Czech Republic and Czechoslovakia), and the pathogen isolates used. Accessions originated from 70 countries (Table S2); more than 100 accessions originated from 11 of them. The highest number of accessions came from Germany (363), including those from the former German Democratic Republic, whereas fewer than four accessions originated from 21 countries. The origin of 61 accessions was unknown.

The response frequency observed in accessions after inoculation is shown in Table S3. The set of 25,236 responses is sorted into 14 IRs, nine of which were homogeneous (0–4 including intertypes) and five of which were heterogeneous. Resistance comprised six homogeneous (0 up to 2–3) and three heterogeneous IRs (0+, 1+, and 2+) and was represented by 15,871 (62.9%) responses. The remaining three homogeneous (3, 3–4, and 4) and two heterogeneous IRs (3+ and 4+) included 9365 (37.1%) susceptible responses of accessions to the individual isolates. In total, 2420 (9.6%) IRs were heterogeneous; in 482 accessions, only one heterogeneous IR was found, whereas in one accession (Coast), all nine IRs were heterogeneous (Table 1).

The resistance of an accession to an isolate has a binary character, which, in the case of the nine isolates, allowed 2^9^ = 512 IRAs to be resolved; in all tested accessions, 266 of the IRAs were found (Table S4). The most abundant was IRA 000 with the highest resistance complexity of nine, reflecting resistance in 627 accessions to all the isolates used. Each of the other three IRAs (100 and 200 with a resistance complexity of eight, and 300 with a resistance complexity of seven) included more than 100 accessions, while each of the 98 IRAs was found only in one accession. The most frequently observed IRAs had resistance complexities of five (60) and four (57), but the most frequent accessions were those with the highest resistance complexities of nine (627 accessions described above), eight (421), and seven (381). The IRA 777 included 143 accessions with a resistance complexity of zero (i.e., they were susceptible to all nine PM isolates), and the IRA 773 included 304 accessions with a resistance complexity of one. Sixty-one IRAs included 320 accessions with resistance complexity ≤3 (Table 2). Accessions with the lowest complexity of resistance provided by major genes are the most suitable for achieving the goal of this research.

## 3. Discussion

The aim of this study was to detect major resistance genes to PM in a large set of diverse spring barley GB accessions. The selection of nine isolates of the pathogen with the narrowest virulence spectra was utilized for this goal and allowed the detection of all known [6,7] and unknown resistance genes. In Tables S1 and S3, isolates are ranked in ascending order by complexity of avirulence, reflecting the complexity of crop resistance to the isolates. Two domestic isolates originated from the area of the highest diversity (central Europe), as well as the highest complexity of virulence of the pathogen [10]. These isolates were characterized by the narrowest spectra of avirulence (the lowest ability to detect major genes) and showed 1300 and 1321 resistant IRs. Conversely, the narrowest spectrum of virulence was recorded for the Japanese isolate Race I, which detected 2431 resistance IRs, with the largest difference between the two European isolates and Race I, especially in the IR0 category (570 and 594 versus 1833 IRs) (Table S3).

The narrowest virulence spectra of the isolates resulted in 143 (5.1%) accessions with no specific resistance and classified as IRA 777 (Table S1). Another IRA 773 comprised 304 accessions carrying resistance genes *Mla8* or *MlCh*. Avirulence to the first of them (IR0 or 0–1) was found only in some Japanese isolates many decades ago [29], and such isolates are probably no longer present in the field. Therefore, accessions carrying *Mla8* cannot control powdery mildew in the field. *MlCh* (typical IR2 or 2–3) is probably a similar example because it can be detected only with the same isolate (Race I) as *Mla8*. Thus, infection of these three groups of accessions (i.e., with the absence of any specific resistance and with the presence of *Mla8* or *MlCh*) cannot be modified by these two named or non-existent specific major genes, and all 447 (143 + 304) accessions are suitable for recovering quantitative minor resistance genes.

In our laboratory, we have been characterizing the inauthenticity of accessions maintained in spring barley genebanks [30,31]. This is a serious problem about which there has been no reliable information available, and it is especially relevant for all cereals. The use of inauthentic accessions leads to false results that invalidate conclusions and lead to a lack of repeatability in subsequent research. In breeding, the use of inauthentic accessions cannot successfully combine particular genes in new varieties. Since there were a limited number of isolates that could be used for the present study, and because of their selection, it was not possible to postulate resistance genes in most of the tested accessions and thus to confirm the presence or absence of these genes in varieties previously studied. However, some cases indicate discrepancies between published resistance genes and current results and highlight the problem of inauthenticity of accessions.

As examples, the varieties Roxana, Explorer, Grace, Kangoo, Pioner, Sunshine, and Gesine, for which Ro resistance, designated according to the name of the first-named variety [32], was identified in registration trials. The identity or similarity of their IRAs obtained here confirmed or at least did not contradict this finding, except for Gesine. This variety and sunshine had been studied together, and an identical resistance conditioned by three genes (*MlRo*, *MlLa,* and *MlCh*) was established in both of them [33]. However, in the present report, the IRAs of these varieties differed significantly (Table S1), thereby proving the inauthenticity of Gesine.

In the previously mentioned catalog [8], the Ar resistance was present in Irania, Luna, Mirena, Nebi, and Nudinka. The identical IRAs of the first four varieties confirmed the presence of Ar, whereas Nudinka showed a different IRA, indicating the accession’s inauthenticity. Similarly, the presence of the *Mla8* resistance gene has been reported in Carlsberg II, Kneifel, Opal B, Siri, Vollkorn Probsdorfer, Kenia Abed, and Mari Svalofs. In the first five, this gene is confirmed by the IRAs obtained here, while the IRAs of the last two varieties differed significantly, and it is probable that *Mla8* is absent in both of them.

Our current goal is not to detect inauthentic accessions, but to reveal accessions that can be exploited easily in breeding modern varieties [34] with accumulated (pyramided) minor PM resistance genes. Therefore, despite the fact that major resistance genes were found in earlier studies in accessions, such as Mazurka (*Mla7*, *Mlg*), Fox (*Mla7*, *Mlk*, *MlLa*) [8] or Engledow India [6], they can also be used for the present purpose because the accessions tested here did not contain any detected major resistance (Fox) or *Mla8*. Conversely, Kristina, Morgenrot, and Sladar do not contain any major resistance genes [8], but in our tests, the IRAs obtained from these accessions showed the presence of major resistance genes, indicating their inauthenticity and unsuitability for the above-mentioned use.

There are few references to detecting minor resistance genes to barley powdery mildew and not much more to powdery mildew of wheat. Under field conditions, Czembor et al. [35] evaluated resistance against powdery mildew, brown rust, and stem rust in 431 European barley accessions, and for mildew resistance, they identified five marker–trait associations. Similarly, the phenotype of powdery mildew resistance was evaluated on a set of 234 recombinant inbred wheat lines derived from the cross of resistant CM104 (Chuanmai 104) and susceptible Baimaomai [36], evaluated in five environments. Three QTLs were identified on chromosomes 7D and 4A; two were minor, and one was a major locus for adult-plant resistance. A recombinant inbred line population, obtained by crossing bread wheat cultivars Victo and Spada, was evaluated for resistance to leaf rust and powdery mildew at the seedling stage [37]. One major QTL was detected in response to mildew on chromosome 7A, which explained 90% of phenotypic variation.

Shikha et al. [38] summarized a wide spectrum of literature and analyzed components and parameters of slow disease development in crops, such as the area under progress curve, disease severity assessed as the percentage of the given plant part (mainly leaves) covered by disease symptoms, infection frequency, including rate between the number of spoors and the number of developed colonies, infection rate (the number of spoors produced per colony or per unit area), incubation or latent period and others. However, very little is known about them and about their individual influence on disease development, especially in the case of powdery mildews.

Crop resistance to diseases, as with many other traits, is based on qualitative and quantitative genes—the latter often controlled by multiple factors with a small effect (quantitative trait loci—QTLs), whose combinations usually result in partial durable resistance [39,40,41,42,43] subject to Mendelian genetics. Quantitative resistance conditioned by minor genes and protecting against all pathotypes has usually been more durable than resistance based on major genes effective only against avirulent strains of the pathogen [44], although some OTLs may be specific [45]. It is more difficult to use polygenic quantitative resistance in breeding, but rapid progress in sequencing technologies shows promise for achieving this aim [46].

## 4. Materials and Methods

### 4.1. Plant Material and Pathogen Isolates

The Czech genebank (GB) of spring barley was used in these investigations. This collection contains almost 3.5 thousand accessions, of which 2804 were tested in the present study (Table S1). Another 223 accessions included in the GB core collection had been assessed in more detail earlier [30]. Results are omitted from the 59 accessions carrying resistances derived from wild barley (*Hordeum vulgare* subsp. *spontaneum*) or bulbous barley grass (*Hordeum bulbosum* L.), which have shown resistance to all or almost all isolates, and the results are also not presented for the newest accessions or for accessions with incomplete results because of low seed germination.

For resistance tests, nine selected reference isolates of *Blumeria hordei* were used, which had been collected from all six non-polar continents over a period of 63 years (1953–2016) and represent the global avirulence diversity of the pathogen. Two isolates were European (Czech), three were Asian, including the oldest isolate found in Japan in 1953 [29], a Chinese isolate, and an Israeli isolate collected on wild barley. Each of the remaining isolates represented one of the four remaining continents. Their responses to 15 standard barley genotypes carrying different specific resistance genes are shown in Table 3. Before inoculation, all isolates were checked for their purity, and their correct pathogenicity phenotypes were verified on standard barley lines [47]. The isolates were multiplied on leaf segments of the susceptible variety Bowman.

### 4.2. Preparation of Barley Accessions and Their Inoculation

About 20 seeds of each accession were sown in a pot (80 mm diameter) filled with a gardening peat substrate and placed in a PM-proof greenhouse under natural daylight. Central leaf segments of 15 mm were cut from fully expanded primary leaves. Three segments of each accession were placed on the surface of a medium consisting of 0.8% water agar containing 40 mg-L of benzimidazole—a leaf senescence inhibitor —in a 150 mm Petri dish. Leaf segments were placed adjacent to each other, along with four segments of Bowman oriented diagonally with their adaxial side facing upward. Each dish usually contained 26–30 tested accessions (78–90 leaf segments).

For inoculation, a cylindrical metal settling tower of 150 mm in diameter and 415 mm in height was used, and a dish with leaf segments was placed at the bottom of the tower. Conidia of each isolate taken from a leaf segment of the susceptible cultivar with fully developed pathogen colonies were shaken onto a square piece (40 × 40 mm) of black paper to visually control the amount of inoculum deposited. Then the paper was rolled to form a blowpipe, and conidia of the isolate were blown through a side hole of 13 mm diameter in the upper part of the settling tower over the Petri dish at a concentration of ca. 10 conidia mm^−2^. The dishes with inoculated leaf segments were incubated at 20 ± 1 °C under artificial light (cool white fluorescent lamps providing 12 h light at 30 ± 5 μmol m^−2^ s^−1^). Before inoculation with another isolate, work tools, including the inoculation tower and the surface of the laboratory table, were sterilized by wiping with cotton wool moistened with 96% ethyl alcohol.

### 4.3. Evaluation and Variety Response Classification

**Figure 2:**
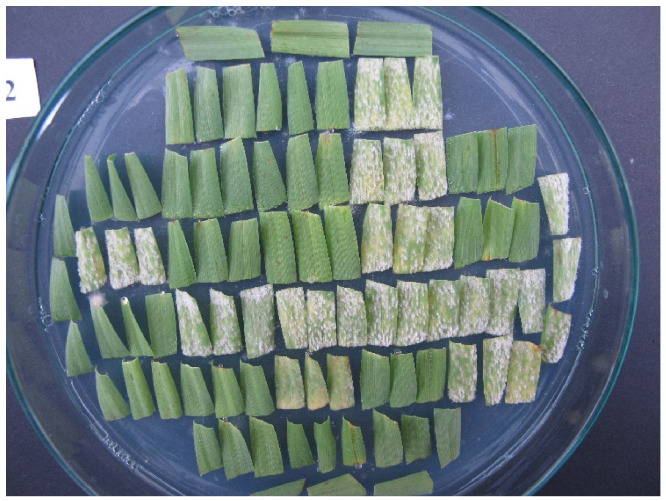
Petri dish with triplets of leaf segments of barley lines carrying effective and ineffective major resistance genes against powdery mildew caused by *Blumeria hordei*.

Seven days after inoculation, the infection response (IR = phenotype of accession × isolate interaction) on the middle part of the leaf segments was scored on a nine-point scale, 0–4, including intertypes (Table 4), where 0 = no mycelium and sporulation, and 4 = strong mycelial growth, strong sporulation, and no chlorosis or necrosis [48]. Homogeneous IRs from 0 up to 2–3 were considered resistant, and IRs 3, 3–4, and 4 were susceptible (Figure 2). Mixed IRs (x + x, where x is 0–4) were heterogeneous and classified as resistant or susceptible according to the first and more frequent IR. During phenotyping, special attention was paid to the boundary IRs 2–3 and 3, which pose the greatest risk of error in distinguishing between virulence and avirulence. Each accession was scored twice, and if there were significant differences between the scorings, an additional evaluation or additional tests were performed. A set of nine IRs provided an infection response array (IRA) for each accession.

Numerical classification of variety IRAs, sometimes referred to as resistance spectra, was based on their resistance/susceptibility patterns to the set of nine isolates ranked in the order shown in Table S1 and divided into three triplets. Each of the digits indicates susceptibility to the three isolates of the respective triplet. If susceptibility to a corresponding isolate was detected, the first isolate is assigned a value of 1 (2^0^), the second a value of 2 (2^1^), and the third a value of 4 (2^2^). Therefore, each digit has a value from 0 (no susceptibility to any of the three isolates) up to 7 (=1 + 2 + 4) denoting susceptibility to each of a triplet of isolates. The resulting number (reverse-octal) defines the IRA identification [49]. In the case of a heterogeneous IR, the first IR was used for constructing the corresponding digit. Details of the method have been recently described, and demonstration images presented [50].

## Figures and Tables

**Table 1:** Number of heterogeneous infection responses recorded on spring barley accessions after inoculation with nine powdery mildew isolates, number of heterogeneous accessions and total number of heterogeneous responses.

Number of Heterogeneous Infection Responses Found in One Accession	Number of Heterogeneous Accessions	Total Number of Heterogeneous Responses
1	482	482
2	316	632
3	180	540
4	94	376
5	46	230
6	19	114
7	3	21
8	2	16
9	1	9
Σ	1143	2420

**Table 2:** Resistance complexity of spring barley accessions, number of infection response arrays (IRAs), and number of accessions.

Resistance Complexity	Number of IRAs	Number of Accessions
0	1	143
1	7	332
2	14	132
3	41	161
4	57	180
5	60	176
6	45	251
7	31	381
8	9	421
9	1	627
Σ	266	2804

**Table 3:** Infection response arrays produced by nine isolates of *Blumeria hordei* on 15 standard barley varieties.

Barley	*Ml*	CZE ^1^	CZE	USA	URG	ISR	CHI	RSA	AUS	JAP
Variety	Gene(s)	P-512	Z-6	MN-b	54	J-462	3-33	65	GH	Race1
		2001	2014	2016	2005	1979	2003	2004	2005	1953
Bowman	*none*	4	4	4	4	4	4	4	4	4
P01 ^2^	*a1*, *aAl2*	0	4	0	0	4	0	0	0	2
P02	*a3*	1	4	1	1	4	1	1	1	1
P03	*a6*, *a14*	4	4	0	0	4	0	0	0	0
P04B	*a7*, *aNo3*	4	4	4	1-2	0-1	0	1-2	1-2	0
Pallas	*a8*	4	4	4	4	4	4	4	4	0
P08B	*a9*	4	0	0	0	0	0	0	0	0
P10	*a12*, *aEm2*	4	4	1	1	4	1	1	1	1
P11	*a13*, *aRu3*	0	0	4	0	0	0	0	0	0
P17	*k*, *a8*	4	2	4	4	4	2	4	4	0
P20	*at*, *a8*	2	2	2	2	4	2	4	4	0
P21	*g*, *a8?*	4	4	0	4	4	0	0	4	0
P22	*mlo*, *a8?*	0	0	0	0	0	0	0	0	0
P23	*La*, *a8*	4	4	4	4	4	2-3	2-3	2-3	0
Kangoo	*Ro*, *a8*	0	4	0	0	4	0	0	0	0

^1^ AUS Australia, CHI China, CZE Czech Republic, ISR Israel, JAP Japan, RSA Republic of South Africa, USA Minnesota, URG Uruguay. ^2^ P = Near-isogenic Pallas line [47].

**Table 4:** Infection responses developed on the adaxial side of barley leaves after inoculation with powdery mildew.

Infection Response	Mycelium Growth	Sporulation	Development of Chlorosis/Necrosis
0	None	None	−
0–1	None	None	+
1	Weak	None	+
1–2	Weak	Weak	+
2	Moderate	Weak	+
2–3	Moderate	Moderate	+
3	Strong	Moderate	+
3–4	Strong	Strong	+
4	Strong	Strong	−

According to Torp et al. [48].

## Data Availability

All final data generated or analyzed during this study are included in this article and its set of four Supplementary Tables. The original raw phenotypic data are not publicly available because of frequent inconsistencies once the first obtained data (IRAs) were subsequently corrected by the results of additional tests, and also owing to the incompleteness of additional tests, when only selected pathotypes were sufficient to confirm or refute the hypothesis about the resistance genes of the tested genotypes. Original raw phenotypic data are available from the corresponding author on reasonable request.

## Supplementary Materials

The following supporting information can be downloaded at: https://www.mdpi.com/article/10.3390/plants15131989/s1, Table S1. Infection response arrays (IRAs) of the Czech genebank spring barley accessions recorded after inoculation with nine powdery mildew isolates; Table S2. Number of spring barley accessions according to their country of origin; Table S3. Number of infection responses recorded on spring barley accessions after inoculation with nine powdery mildew isolates; Table S4. Two hundred and sixty-six infection response arrays (IRAs) coded in reverse octal numbers, their frequency, and the complexity of resistance.
